# A direct fluorescence assay for quantitative analysis of ornithine decarboxylase activity and inhibitor screening

**DOI:** 10.1016/j.jbc.2026.111468

**Published:** 2026-04-17

**Authors:** Jae-Yeon Choi, Grzegorz Bereta, Matthew Furry, Christopher D. Vanderwal, Przemysław Grudnik, Choukri Ben Mamoun

**Affiliations:** 1 Department of Internal Medicine, Section of Infectious Diseases, New Haven, Connecticut, USA; 2 Małopolska Centre of Biotechnology, Jagiellonian University, Kraków, Poland; 3 Department of Chemistry, University of California, Irvine, California, USA; 4 Department of Pharmaceutical Sciences, University of California, Irvine, California, USA; 5 Department of Microbial Pathogenesis, New Haven, Connecticut, USA; 6 Department of Pathology, Yale School of Medicine, New Haven, Connecticut, USA

**Keywords:** 1,2-diacetyl benzene, cancer, drug discovery, enzyme activity, fluorescence assay, inhibition, isoindole, ornithine, ornithine decarboxylase, polyamines, putrescine, spermidine, spermine

## Abstract

Ornithine decarboxylase (ODC) catalyzes the first committed step in polyamine biosynthesis and plays a central role in cellular growth and proliferation. Quantitative analysis of ODC activity has traditionally relied on radiometric or coupled-enzyme assays, which limit scalability and accessibility. Here, we report on the development of 1,2-diacetylbenzene (DAB)-ODC, a fluorescence-based assay that enables direct, sensitive, and high-throughput quantification of ODC activity by detecting putrescine through its reaction with DAB. Using purified recombinant ODC from *Saccharomyces cerevisiae* and humans, we show that DAB-ODC supports measurement of enzyme activity and accurate determination of steady-state kinetic parameters. Inhibition studies with the inhibitor DL-α-difluoromethylornithine and three recently reported DL-α-difluoromethylornithine analogs yielded IC_50_ values consistent with those obtained using established orthogonal assays. All enzymatic measurements were validated by thin-layer chromatography. Assay performance metrics, including CV, Z′ factor, and signal-to-background ratios, demonstrate compatibility with large-scale chemical screening. Together, these results establish DAB-ODC as a versatile platform for ODC enzymology, inhibitor profiling, and high-throughput interrogation of polyamine metabolism.

Polyamines are low-molecular-weight aliphatic amines essential for cellular growth and survival (1, 2). Structurally characterized by their multiple amine groups, they are biologically crucial due to their strong positive charge, which allows them to interact electrostatically with negatively charged molecules such as DNA, RNA, and proteins (3, 4, 5, 6). These interactions are vital for stabilizing DNA structures, regulating ion channels, modulating enzyme activities, and facilitating signal transduction pathways involved in cell cycle progression and cellular differentiation (7).

The primary polyamines in eukaryotic cells, putrescine, spermidine, and spermine, are synthesized through a highly conserved, stepwise biosynthetic pathway (Fig. 1*A*) and play distinct and non-redundant roles in cellular physiology (4). Putrescine, the simplest polyamine, is pivotal in initiating cellular proliferation and modulating cellular signaling pathways (4). Spermidine is extensively involved in autophagy and the modulation of transcriptional and translational processes, whereas spermine, the most complex of the three, is particularly crucial for the stabilization of cellular macromolecular complexes and maintaining chromatin structure in a state conducive for transcription (8). The polyamine biosynthesis pathway has been widely recognized as a promising target for developing novel therapies against microbial infections, cancer, and neurodegenerative diseases (9, 10, 11, 12, 13).

**Figure 1:**
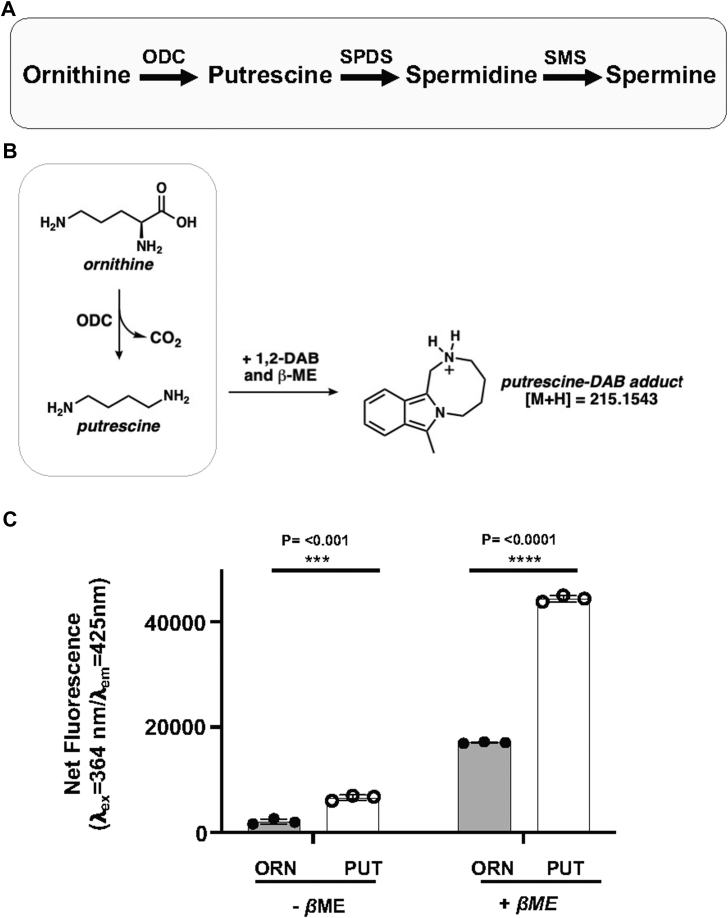
**Chemical basis and quantitative performance of the DAB-based assay for detection of ornithine decarboxylase activity.***A*, schematic overview of the polyamine biosynthesis pathway showing conversion of ornithine to putrescine by ornithine decarboxylase (ODC), followed by synthesis of spermidine and spermine through spermidine synthase (SPDS) and spermine synthase (SMS), respectively. *B*, reaction scheme illustrating selective derivatization of putrescine by DAB in the presence of βME, generating a stable fluorescent adduct used as the readout of ODC activity. The chemical structures of ornithine, putrescine, and the putrescine–DAB fluorescent adduct are shown. *C*, ornithine and putrescine (250 μM each) were prepared in 1 mM Tris-HCl (pH 7.5), and 17.5 μl was used for DAB derivatization and fluorescence detection as described in the Experimental procedures. Reactions were performed with DAB in the absence or presence of βME, and fluorescence was measured at λ_ex_= 364 nm and λ_em_= 425 nm. Putrescine produced a markedly stronger fluorescence signal than ornithine, and βME enhanced overall fluorescence intensity. Statistical significance was assessed by unpaired two-tailed *t* test. The data are presented from three independent experiments performed in triplicates, and values are mean ± S.D. βME, B-mercaptoethanol; DAB, 1,2-diacetylbenzene; ODC, ornithine decarboxylase

Putrescine synthesis is catalyzed by the enzyme ornithine decarboxylase (ODC), which decarboxylates ornithine derived from the urea cycle (14, 15). This reaction is the first and rate-limiting linking nitrogen metabolism to downstream polyamine production. In humans, ODC is encoded by *ODC1* (10), whereas in fungi the orthologous enzyme is encoded by *SPE1* (16), which is conserved across multiple fungal species. In eukaryotes, the polyamine biosynthetic pathway downstream of ODC/Spe1 is conserved in its core enzymatic steps but diverges in how polyamines are integrated into broader metabolic networks. In *Saccharomyces cerevisiae*, Spe1 catalyzes the committed step into polyamine biosynthesis, producing putrescine that is subsequently converted to spermidine and spermine, which contribute to diverse cellular processes including protein translation, autophagy, redox balance, and lipid metabolism. In fungi, spermine additionally serves as a precursor for pantothenic acid (vitamin B5) biosynthesis, linking polyamine metabolism to coenzyme A homeostasis. In human cells, ODC catalyzes the rate-limiting step of polyamine biosynthesis, and polyamines are implicated in diverse cellular processes, including gene regulation, protein translation, autophagy, cell proliferation, apoptosis, necrosis, and cell reprogramming. ODC’s activity hinges on its interaction with the cofactor pyridoxal 5′-phosphate, forming a Schiff base that facilitates the decarboxylation of ornithine to generate putrescine (17). This enzyme's regulation is multifaceted, involving post-translational modifications, feedback inhibition by polyamines, and transcriptional regulation by antizyme inhibitor, reflecting its central role in cellular proliferation and differentiation (18). Precise quantification of ODC activity is therefore important for mechanistic studies and for identifying new small-molecule inhibitors.

Quantifying ODC activity remains technically challenging because neither ornithine nor putrescine possesses intrinsic optical properties, requiring conversion into a detectable form. As a consequence, several established assays, including radiometric CO_2_-release formats (19, 20), colorimetric and fluorescence derivatization chemistries (21, 22), and chromatographic detection (23), require additional reaction steps, specialized equipment, or discontinuous workflows. These features limit their practicality for medium- or high-throughput applications, where robustness, continuous readout, and minimal manual intervention are essential. Enzyme-coupled systems have also been developed to amplify the ODC signal, most notably using phosphoenolpyruvate carboxylase and malate dehydrogenase as auxiliary enzymes (24). While effective, these cascades inherently depend on the performance of the coupling enzymes and their cofactors, increasing susceptibility to interference from screened compounds and complicating assay optimization. As a result, despite the biological importance of ODC and extensive interest in ODC inhibition, large-scale screening efforts remain limited.

Recent studies have expanded the analytical applications of 1,2-diacetylbenzene (DAB) as a versatile chemical probe for interrogating enzymatic reactions across distinct biological pathways (25, 26, 27, 28). In the context of polyamine metabolism, DAB has been developed into a robust fluorescence-based assay for aminopropyltransferase (APT) activity, where it reacts with enzymatically generated polyamines to form fluorescent isoindole-type adducts, enabling direct measurement of enzyme kinetics, inhibitor profiling, and compatibility with high-throughput screening formats (26, 27, 28). It was shown that the fluorescence signal generated by DAB correlates strongly with the chemical structure of the polyamine, increasing with polyamine chain length and degree of substitution, such that spermidine and spermine yield substantially higher fluorescence than putrescine under identical reaction conditions. This property was exploited to sensitively quantify APT-mediated aminopropyl transfer reactions, in which conversion of putrescine to higher-order polyamines produces a marked increase in fluorescence signal (28). Beyond polyamine metabolism, DAB has also been applied as a chemical tool to examine enzymatic activity in phospholipid metabolism, most notably in assays of phosphatidylserine decarboxylase (PSD) (25). In these systems, DAB reacts preferentially with phosphatidylethanolamine (PE), the product of the PSD reaction, which contains a free primary amine, whereas the substrate phosphatidylserine bears an α-amino acid head group that is not reactive under the same conditions (25). Consequently, the DAB–PE adduct generates a substantially higher fluorescence signal while there is no fluorescence generated from interaction of DAB with phosphatidylserine, enabling sensitive, non-radioactive detection of PSD activity through direct monitoring of PE formation (25). Collectively, these studies highlight the unique reactivity, simplicity, and tunable fluorescence output of DAB, supporting its broader utility as a general probe for enzymatic processes that generate or modify primary aliphatic amines, and suggesting that additional biological applications are likely to emerge.

To enable a simple, direct, and automation-compatible readout for ODC activity, we evaluated DAB as a fluorogenic derivatization reagent to differentiate between the substrate and product of the ODC reaction. We leveraged the unique reactivity of DAB together with the distinct chemical structures of the ODC substrate L-ornithine and its product putrescine to develop a novel fluorescence-based assay for ODC activity. Whereas ornithine contains an α-amino acid moiety with reduced reactivity toward DAB, the decarboxylation product putrescine exposes a free primary aliphatic amine that readily forms a highly fluorescent DAB adduct. This reaction proceeds in a single step, requires no auxiliary enzymes or coupled reactions, and produces a stable fluorescence signal suitable for continuous monitoring. The simplicity and robustness of the DAB–putrescine reaction provide a practical foundation for microplate-based kinetic analyses and future high-throughput screening applications. Because the DAB–putrescine reaction is insensitive to dimethyl sulfoxide (DMSO) at concentrations typically used for screening chemical libraries, the assay is readily adaptable to microplate-based formats and provides a practical foundation for future high-throughput identification of ODC inhibitors.

## Results

### Comparison of DAB-dependent fluorescence of ornithine and putrescine

DAB reacts with primary amines to form fluorescent adducts, but this reactivity is strongly influenced by the local chemical environment of the amine. In particular, primary amines whose reactivity is attenuated by an α-carboxyl group show no fluorescent adduct formation, presumably owing to the electron-withdrawing nature of that carboxyl group (25, 29). This differential reactivity has previously enabled DAB-based fluorescence detection of enzymatic activities such as amino acid decarboxylases and phosphatidylserine decarboxylases (25, 29). Ornithine, the substrate of ODC, presents an informative structural case in this context. Ornithine contains two primary amines: one primary amine attached to an α-carbon that bears a carboxyl group, and a second primary amine on the distal side chain that is not associated with a carboxylated carbon. In contrast, the product of the ODC reaction, putrescine, contains two primary amines, neither of which is associated with an α-carboxyl group (Fig. 1*B*). Based on these structural differences, we examined whether ornithine and putrescine exhibit differential fluorescence upon incubation with DAB in the presence or absence of β-mercaptoethanol (βME) using a spectrophotometer with excitation and emission spectra at wavelengths of 364 nm and 425 nm, respectively. Under identical assay conditions, putrescine produced a markedly stronger fluorescence signal than ornithine, with net fluorescence for putrescine measured after 60 min was approximately 3-fold higher than that of ornithine (Fig. 1*C*). The presence of βME substantially enhanced overall fluorescence intensity (Fig. 1*C*).

**Figure 2:**
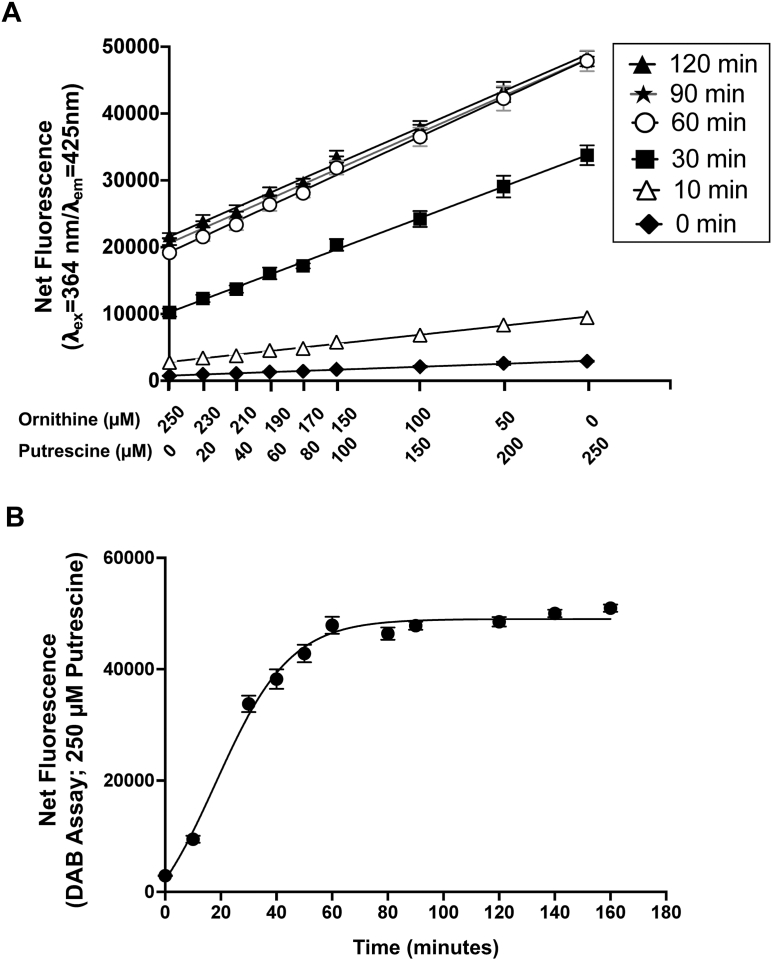
**Putrescine concentration–dependent fluorescence response of the DAB assay.***A*, fluorescence signals generated by DAB adducts formed from mixtures of ornithine and putrescine. Ornithine/putrescine mixtures were prepared under the same buffer conditions used for the ODC enzyme reaction but were not incubated with enzyme and were immediately treated with the DAB fluorescence detection buffer as described in the Experimental procedures. The total combined concentration of ornithine and putrescine was maintained at 250 μM, with nine mixtures containing progressively decreasing ornithine and increasing putrescine concentrations. Increasing putrescine concentrations result in proportional increases in fluorescence, demonstrating that the assay accurately reports conversion of ornithine to putrescine. The slope of the fluorescence response increases over time from 0 to 50 min and remains stable up to 2 h. *B*, time-course analysis of DAB-derived fluorescence from 250 μM putrescine, defining a stable quantitative detection window between approximately 50 min and 2 h. The data are presented from three independent experiments performed in triplicates, and values are mean ± S.D. DAB, 1,2-diacetylbenzene; ODC, ornithine decarboxylase

These data led us to examine whether a DAB-mediated fluorescence assay could be used to measure the activity of ODC enzymes, which catalyze the conversion of ornithine to putrescine (ODC activity). To account for the possibility that different ODC enzymes exhibit distinct ornithine-to-putrescine conversion rates, we mimicked enzyme reaction conditions by performing the DAB assay in the presence of defined mixtures of substrate (ornithine) and product (putrescine) at varying ratios. Fluorescence intensity was measured at different time points and reaction mixtures were analyzed by thin-layer chromatography (TLC) to independently verify the relative contributions of substrate and product to the observed fluorescence signal (Fig. 2*A* and Fig. S1). Under these conditions, DAB-based fluorescence increased in a ratio-dependent manner as the proportion of putrescine increased relative to ornithine (Fig. 2*A*). Mixtures dominated by ornithine consistently produced low fluorescence signals, indicating low reactivity of ornithine with DAB, whereas increases in putrescine concentrations resulted in a concomitant increase in fluorescence. Intermediate substrate–product ratios yielded fluorescence values that scaled with putrescine abundance rather than total amine concentration, demonstrating that the DAB signal primarily reflects product formation. At high putrescine proportions, fluorescence approached a maximal range, consistent with saturation of DAB reactivity. Consistent with these findings, time-course analysis of DAB fluorescence in the presence of putrescine alone (250 μM, no ornithine) showed a rapid increase in fluorescence that reached a stable plateau over time, indicating efficient and reproducible formation of the fluorescent DAB–putrescine adduct and temporal stability of the signal under assay conditions (Fig. 2*B*).

### DAB-based fluorescence measurement of yeast ODC activity catalyzed by ScSpe1

**Figure 3:**
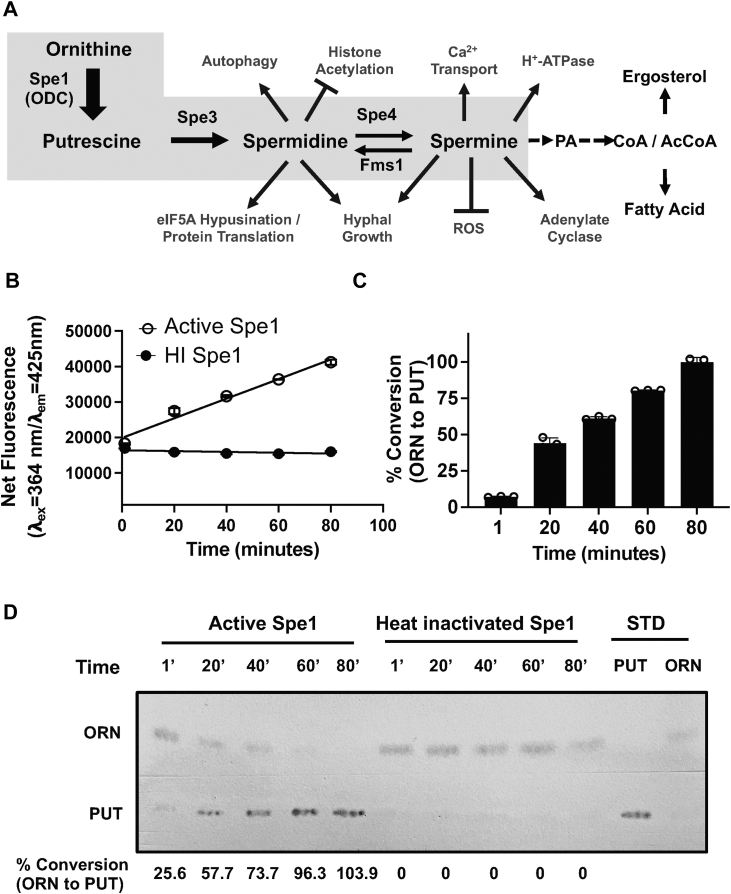
**Polyamine biosynthetic pathway in *Saccharomyces cerevisiae* and validation of ScSpe1 activity by fluorescence and thin-layer chromatography ( TLC)assays.***A*, schematic of the polyamine biosynthetic pathway in *S*. *cerevisiae* and the cellular processes regulated by polyamines downstream of ornithine decarboxylase (ODC/Spe1). Spe1 catalyzes the decarboxylation of ornithine to putrescine, which is subsequently converted to spermidine and spermine. Polyamines regulate multiple cellular functions, including eIF5A hypusination and protein translation, autophagy, redox homeostasis, histone acetylation, ion transport, and lipid metabolism. Unique to fungal species, pantothenic acid (vitamin B5) is synthesized from the polyamine spermine, linking polyamine metabolism to coenzyme A biosynthesis. *B*, time-dependent activity of *S. cerevisiae* Spe1 (ScSpe1) measured using the DAB-based fluorescence assay. The ODC reaction was conducted as described in the Experimental procedures at 0, 20, 40, 60, and 80 min. Increasing reaction time resulted in a corresponding increase in fluorescence, reflecting progressive conversion of ornithine to putrescine by active Spe1. The data are presented from three independent experiments performed in triplicates, and values are mean ± S.D. *C*, percentage conversion of ornithine to putrescine over time, calculated from fluorescence measurements. Conversion increased steadily and reached approximately 100% at 80 min. Percentage conversion was calculated as % conversion = (Ft -F_0_)/(Fmax -F_0_) × 100, where F_t_ is the fluorescence at time t, F_0_ is the background signal, and Fmax corresponds to the signal from complete conversion (putrescine control). *D*, TLC analysis confirming ScSpe1-dependent conversion of ornithine to putrescine. The ODC enzyme reaction mixtures, collected at the indicated incubation times, were resolved by TLC and visualized by ninhydrin staining. Active Spe1 produced increasing putrescine spots with a concomitant decrease in ornithine, whereas heat-inactivated Spe1 showed no detectable putrescine formation. The TLC results closely match the fluorescence-based measurements shown in *panels* B and C. Data are mean ± SD (n = 3 independent experiments, each in triplicate). DAB, 1,2-diacetylbenzene; ODC, ornithine decarboxylase

With the DAB-based fluorescence assay validated, we employed this method to quantitatively assess ODC activity using purified His_6_-tagged ScSpe1 from the yeast *S*. *cerevisiae* by monitoring the enzymatic conversion of ornithine to putrescine (Fig. 3*A*). Using purified ScSpe1, we measured enzyme activity over time by quantifying putrescine formation with the DAB-based fluorescence assay. Incubation of ScSpe1 with ornithine resulted in a time-dependent increase in fluorescence signal, consistent with progressive production of putrescine (Fig. 3*B*). In contrast, no increase in fluorescence could be detected in reactions containing heat-inactivated ScSpe1 (Fig. 3*B*). Fluorescence data were used to calculate the percentage conversion of ornithine to putrescine over time. Conversion increased steadily throughout the reaction and reached approximately 100% by 80 min (Fig. 3*C*), indicating near-complete substrate turnover under the assay conditions. To further validate the fluorescence-based measurements, the same reaction mixtures were analyzed by TLC and visualized by ninhydrin staining. As shown in Figure 3*D*, reactions containing active ScSpe1 showed a time-dependent accumulation of putrescine accompanied by a corresponding reduction in ornithine, whereas heat-inactivated enzyme showed no detectable putrescine formation. The TLC profiles closely matched the fluorescence measurements, confirming that the DAB-based assay accurately reports ScSpe1-catalyzed conversion of ornithine to putrescine. Together, these data demonstrate that purified His_6_-tagged ScSpe1 is catalytically active and that the DAB-based fluorescence assay provides a robust, quantitative, and enzyme-specific readout of ODC activity, suitable for downstream kinetic and inhibitor studies.

### Kinetic characterization and inhibitor validation of yeast ScSpe1 using a DAB-based fluorescence assay

**Figure 4:**
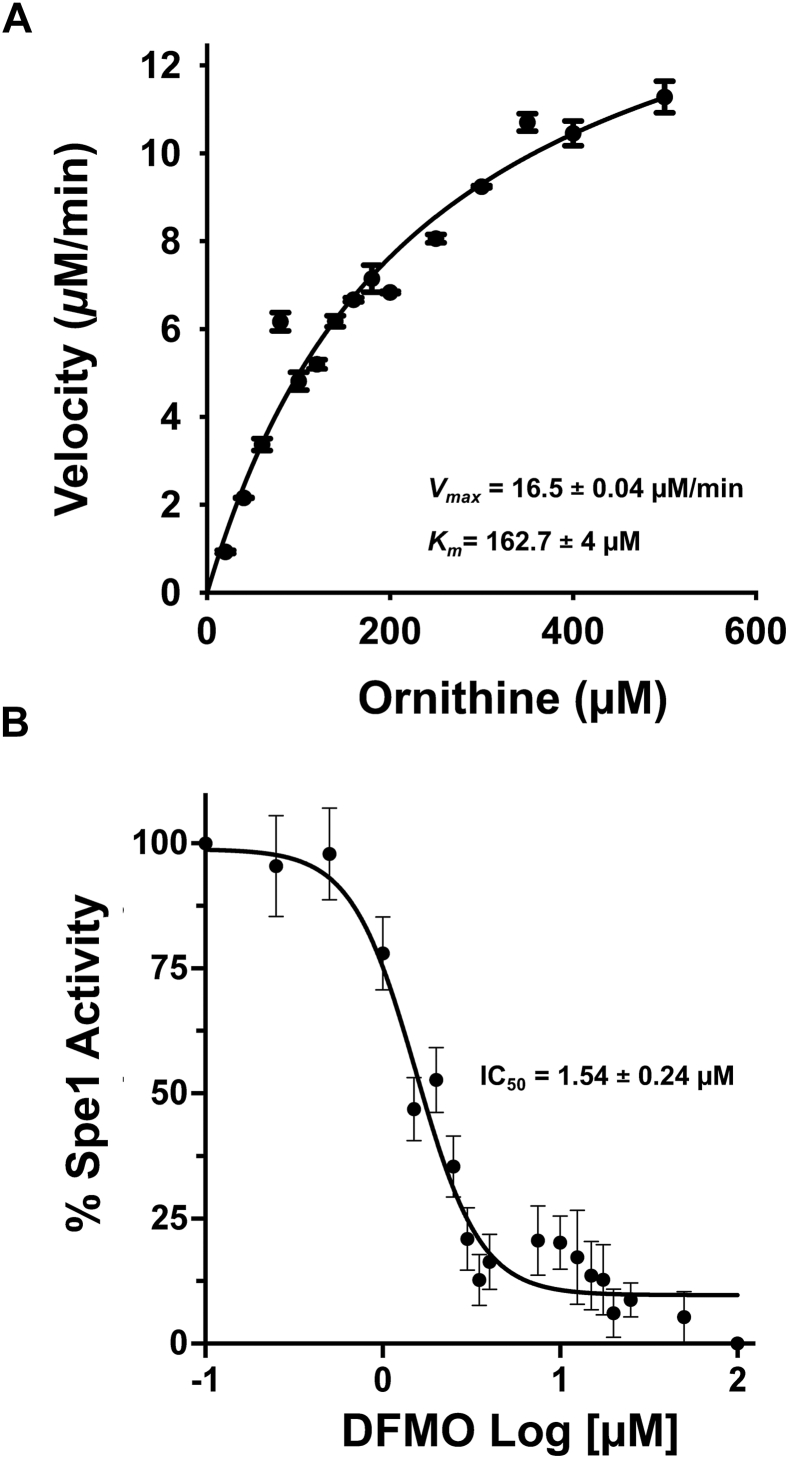
**Kinetic characterization of ScSpe1 and inhibition by DFMO using the DAB-based fluorescence assay.***A*, Michaelis–Menten analysis of *S. cerevisiae* Spe1 (ScSpe1) activity derived from fluorescence measurements shown in Fig. S2*A*. ODC enzyme reactions were conducted for 20 min at ornithine concentrations ranging from 0 to 500 μM, followed by DAB derivatization and fluorescence detection. Reaction velocity was calculated from fluorescence values normalized to the signal corresponding to theoretical 100% conversion (putrescine control). Reaction velocities were plotted as a function of ornithine concentration and fitted using the Michaelis–Menten equation, *v*=*V*_*max*_[S]/(*K*_*m*_+ [S]) where *v* is the initial velocity, and [*S*] is the ornithine concentration and *K*_*m*_ represents the substrate concentration at half-maximal velocity (GraphPad Prism 10). The calculated kinetic parameters were a *V*_*max*_ of 16.5 ± 0.04 μM min^-1^ and a *K*_*m*_ of 162.7 ± 4 μM. *B*, inhibition of ScSpe1 activity by the ornithine analog DFMO. ODC reactions were performed with 160 μM ornithine and incubated for 30 min in the presence of increasing concentrations of DFMO, as described in the Experimental procedures, followed by DAB-based fluorescence detection. Enzyme activity was expressed as a percentage of control activity. Data were fitted by nonlinear regression using a four-parameter logistic equation *Y* = *Bottom*+ (*Top* - *Bottom*)/(1 + ([*I*]/IC_50_)^n^), where *Y* is the normalized response, [*I*] is the DFMO concentration, and n is the Hill slope. Dose-dependent inhibition was observed, yielding an IC_50_ of 1.54 ± 0.24 μM. Data are mean ± SD (n = 3 independent experiments, each in triplicate). DAB, 1,2-diacetylbenzene; ODC, ornithine decarboxylase; DFMO, DL-α-difluoromethylornithine

To determine the kinetic parameters of the yeast ScSpe1, enzyme activity was measured across a range of ornithine concentrations up to 800 μM, and net putrescine-dependent fluorescence was quantified following adduct formation with DAB. Increasing substrate concentrations resulted in a corresponding increase in fluorescence signal, which approached saturation at approximately 250 μM ornithine (Fig. S2*A*), consistent with enzyme saturation under these assay conditions. Reaction velocities calculated from the fluorescence data were plotted as a function of ornithine concentration and fitted to the Michaelis–Menten equation using nonlinear regression (GraphPad Prism: https://www.graphpad.com/scientific-software/prism). This analysis yielded a *V*_*max*_ of 16.5 ± 0.04 μM min^−1^ and a *K*_*m*_ of 162.7 ± 4 μM for ScSpe1 (Fig. 4*A*), indicating moderate substrate affinity and robust catalytic activity of the purified enzyme.

To validate the DAB-based assay for inhibitor studies, we examined the effect of the ornithine analog DL-α-difluoromethylornithine (DFMO) on ScSpe1 activity. Increasing concentrations of DFMO resulted in dose-dependent inhibition of enzyme activity, as measured by reduced fluorescence signal relative to control reactions (Fig. 4*B*). Nonlinear regression analysis yielded an IC_50_ value of 1.54 ± 0.24 μM, consistent with the known potency of DFMO as an irreversible ODC inhibitor (30). Together, these data demonstrate that the DAB-based assay reliably reports both substrate-dependent kinetics and pharmacological inhibition of ScSpe1.

### Application of the DAB-based fluorescence assay for characterization of human ornithine decarboxylase

**Figure 5:**
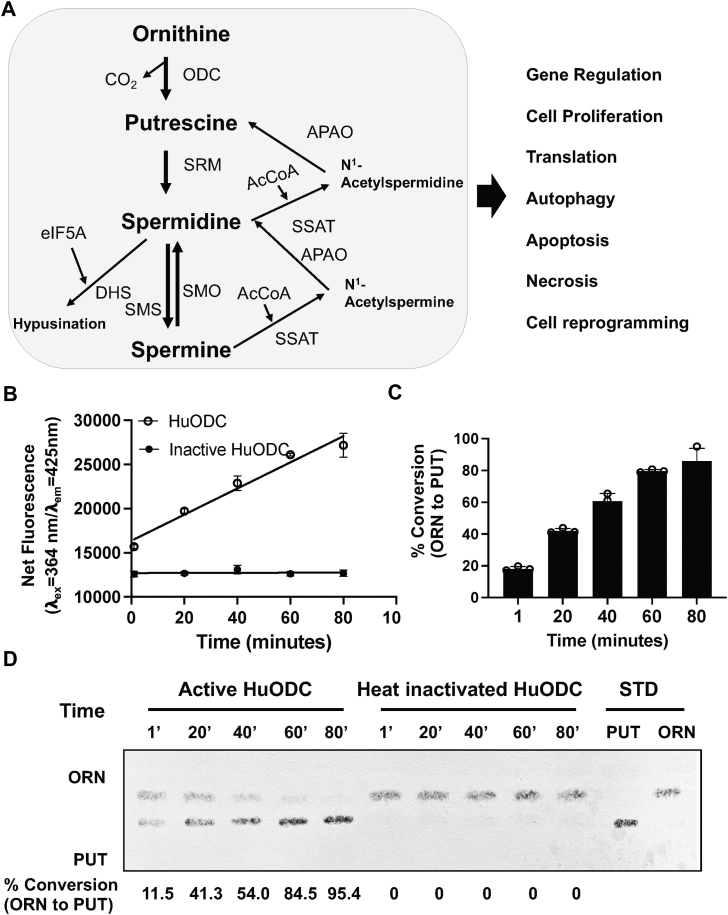
**Measurement of human ODC activity using an MBP–HuODC fusion protein and the DAB-based fluorescence assay.***A*, schematic of the human polyamine biosynthetic pathway downstream of ODC, highlighting roles of polyamines in gene regulation, protein translation, autophagy, cell proliferation, apoptosis, and cell fate decisions. *B*, time-dependent activity of purified maltose-binding protein–fused human ODC measured using the DAB-based fluorescence assay. ODC reactions were conducted under the same conditions used for ScSpe1 in Figure 3*B*, as described in the Experimental procedures, with 250 μM ornithine and incubated for 0, 20, 40, 60, and 80 min, followed by DAB-based fluorescence detection. Fluorescence increased with reaction time, indicating progressive conversion of ornithine to putrescine, analogous to ScSpe1 activity shown in Figure 2*B*. *C*, percentage conversion of ornithine to putrescine over time calculated from fluorescence measurements, reaching near-complete conversion by 80 min, comparable to the behavior observed for ScSpe1 (Fig. 2*C*). Percentage conversion was calculated as % conversion = (F_t_ - F_0_)/(F_max_ -F_0_) × 100, where F_t_ is the fluorescence at time t, F_0_ is the background signal, and Fmax corresponds to the signal from complete conversion (putrescine control). *D*, TLC analysis confirming HuODC-dependent conversion of ornithine to putrescine. Active maltose-binding protein–fused human ODC produced increasing putrescine spots with concomitant decreases in ornithine, whereas heat-inactivated HuODC showed no detectable putrescine formation, consistent with fluorescence-based measurements and analogous to Figure 2*D*. DAB, 1,2-diacetylbenzene; ODC, ornithine decarboxylase; HuODC, human ODC

Because human ODC (HuODC) is a clinically relevant driver of polyamine-dependent proliferation and a therapeutic target in cancer (Fig. 5*A*) (10), we next applied the DAB-based fluorescence assay to quantify HuODC activity. Incubation of recombinant HuODC with ornithine resulted in a time-dependent increase in DAB-derived fluorescence, indicating progressive formation of putrescine (Fig. 5*B*). No increase in fluorescence was observed in reactions containing heat-inactivated HuODC, confirming that the signal was enzyme dependent. Quantitative analysis of fluorescence over time showed a steady increase in the percentage conversion of ornithine to putrescine, reaching near-complete conversion by 80 min under the assay conditions (Fig. 5*C*). TLC analysis of the same reaction mixtures confirmed HuODC-dependent conversion of ornithine to putrescine. Active maltose-binding protein–fused human ODC produced increasing putrescine spots accompanied by a concomitant decrease in ornithine, whereas heat-inactivated enzyme showed no detectable product formation (Fig. 5*D*). These results closely mirrored those obtained with ScSpe1, demonstrating that the DAB-based assay is readily transferable from fungal to human ODC.

### Kinetic characterization and inhibition of human ODC

**Figure 6:**
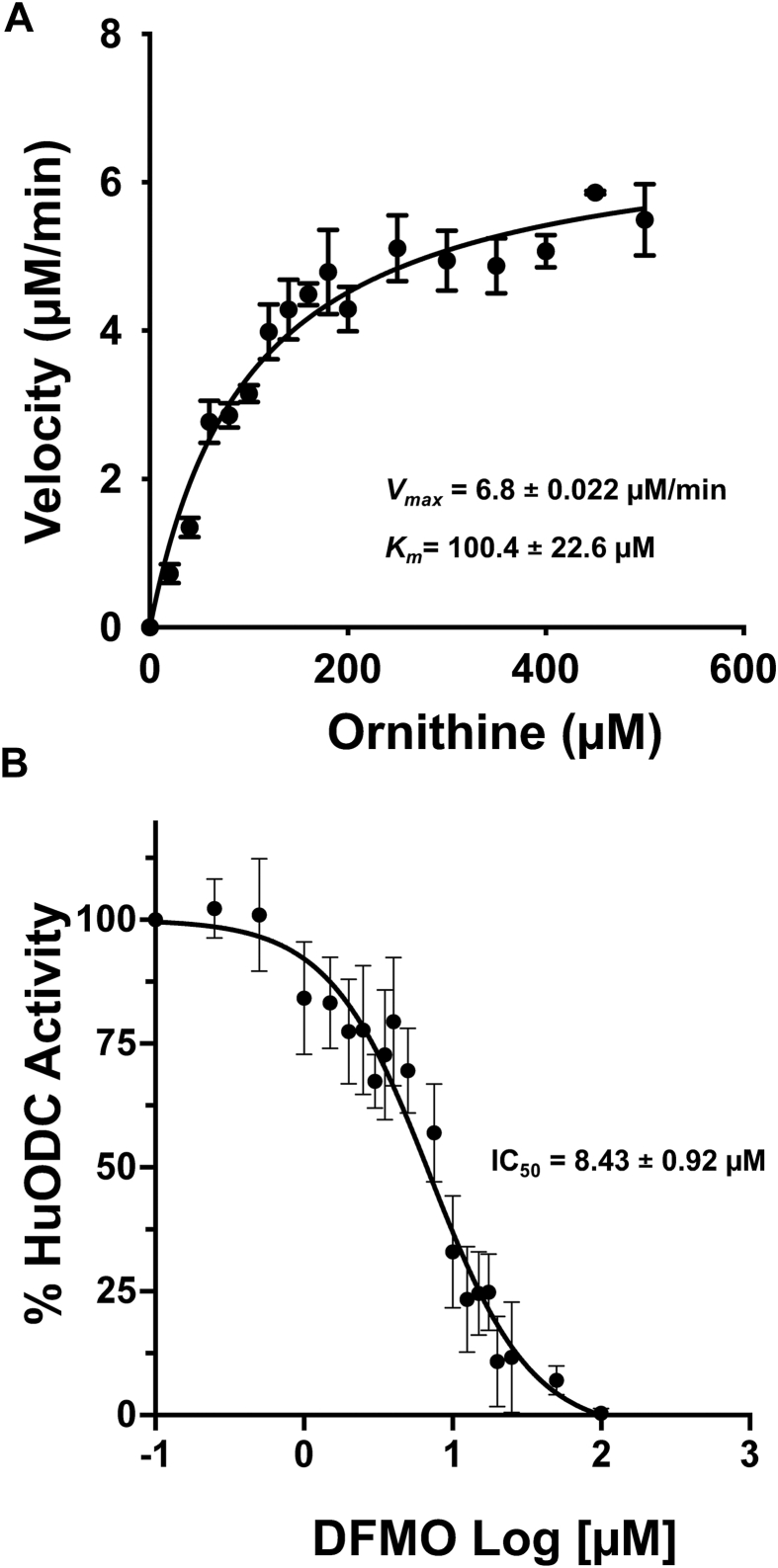
**Kinetic characterization and inhibition of MBP fused recombinant human ODC using the DAB-based fluorescence assay.***A*, Michaelis–Menten analysis of HuODC activity derived from fluorescence measurements shown in Fig. S2*A*. ODC enzyme reactions were conducted for 20 min at ornithine concentrations ranging from 0 to 500 μM, followed by DAB derivatization and fluorescence detection. Reaction velocity was calculated from fluorescence values normalized to the signal corresponding to theoretical 100% conversion (putrescine control). Reaction velocities were plotted as a function of ornithine concentration and fitted by Michaelis–Menten equation, *v*=*V*_*max*_[*S*]/(*K*_*m*_+[*S*]) where *v* is the initial velocity, and [*S*] is the ornithine concentration and *K*_*m*_ represents the substrate concentration at half-maximal velocity (GraphPad Prism), yielding a *V*_*max*_ of 6.8 ± 0.022 μM min^−1^ and a *K*_*m*_ of 100.4 ± 22.6 μM. *B*, inhibition of HuODC activity by DFMO. ODC reactions were performed with 100 μM ornithine and incubated for 30 min in the presence of increasing concentrations of DFMO, as described in the Experimental procedures, followed by DAB-based fluorescence detection. Enzyme activity was measured at increasing DFMO concentrations and expressed as percentage of control activity. Data were fitted by nonlinear regression using a four-parameter logistic equation *Y* = Bottom + (*Top - Bottom*)/(1 + ([*I*]/IC_50_)^n^), where *Y* is the normalized response, [*I*] is the DFMO concentration, and n is the Hill slope. Dose-dependent inhibition was observed, yielding an IC_50_ of 8.4 ± 0.92 μM. Data are mean ± SD (n = 3 independent experiments, each in triplicate). HuODC, human ODC; DAB, 1,2-diacetylbenzene; DFMO, DL-α-difluoromethylornithine

Using the validated fluorescence assay, we next determined the kinetic parameters of HuODC. Net fluorescence increased with increasing ornithine concentrations and approached saturation, indicating enzyme saturation at higher substrate concentrations (Fig. S2*B*). Michaelis–Menten analysis of the fluorescence-derived velocities yielded a *V*_*max*_ of 6.8 ± 0.022 μM min^−1^ and a *K*_*m*_ of 100.4 ± 22.6 μM for ornithine (Fig. 6*A*). The *K*_*m*_ value determined for HuODC is modestly lower than that measured for ScSpe1 and is consistent with reported values for mammalian ODC enzymes, supporting the physiological relevance of the assay-derived kinetic parameters.

To assess suitability of the assay for inhibitor studies, we examined inhibition of HuODC by DFMO. DFMO produced dose-dependent inhibition of HuODC activity as measured by reduced fluorescence signal, yielding an IC_50_ of 8.4 ± 0.92 μM (Fig. 6*B*).

### Validation of the DAB-based ODC assay using non-DFMO inhibitors

In addition to DFMO, we examined three previously reported ODC inhibitors from the study by Schultz and colleagues (31): 1-amino-oxy-3-aminopropane (APA) and the enantiomeric 3-[(aminooxy)methyl]pyrrolidine analogs, hereafter referred to as R-AOMP and S-AOMP (corresponding to 10R and 10S in the original report (31) (Table 1). These compounds have been shown to exhibit substantially higher potency than DFMO (31, 32), providing a stringent test of the DAB-based fluorescence assay for detecting inhibition by chemically diverse ODC inhibitors. APA, R-AOMP, and S-AOMP each reduced ODC activity in both HuODC and ScSpe1 assays in a concentration-dependent manner (Fig. S3). Increasing inhibitor concentrations were associated with progressively lower percent activity relative to the no-inhibitor control, as quantified by the DAB-based fluorescence assay. Using full dose–response datasets, APA potently inhibited ScSpe1 with an IC_50_ of 9 nM and HuODC with an IC_50_ of 16 nM. The stereoisomeric inhibitors R-AOMP and S-AOMP also inhibited both enzymes in a concentration-dependent manner, with IC_50_ values of 33 nM and 547 nM for ScSpe1, and 136 nM and 1400 nM for HuODC, respectively (Table 1). Together, these data demonstrate that the DAB-based fluorescence assay provides a robust and quantitative platform for measuring human ODC activity, determining kinetic parameters, and evaluating pharmacological inhibition, enabling direct comparison of enzymatic properties between fungal and human ODCs.

**Table 1:** Inhibition of *S*. *cerevisiae* Spe1 and human ODC by DFMO and non-DFMO ODC inhibitors

Inhibitor	Structure	MW (Da)	*S. cerevisiae* Spe1		Human ODC	
			IC_50_ (μM)	% inhibition at 5 μM	IC_50_ (μM)	% Inhibition at 5 μM
DFMO		182	1.54 ± 0.24	87.3	8.4 ± 0.92	30.5
APA		90	0.009 ± 0.0003	100	0.016 ± 0.001	100
R-AOMP (10-R)		116	0.033 ± 0.002	100	0.136 ± 0.06	100
S-AOMP (10-S)		116	0.547 ± 0.12	95.4	1.4 ± 0.22	87.7

IC_50_ values were determined from dose–response analyses of ScSpe1 and HuODC activity measured using the DAB-based fluorescence assay, as described in the Experimental procedures and shown in Fig. S3. Data were fitted by nonlinear regression using a four-parameter logistic equation *Y* = Bottom + (Top - Bottom)/)/(1 + ([*I*]/IC_50_)^n^), where *Y* is the normalized response, [*I*] is the inhibitor concentration, and n is the Hill slope. Percent inhibition values represent enzyme activity remaining at 1 μM inhibitor, normalized to no-inhibitor controls. ODC reactions were performed with 160 and 100 μM ornithine for ScSpe1 and HuODC, respectively and incubated for 30 min prior to heat inactivation, DAB derivatization, and fluorescence detection. Data are mean ± SD (n = 3 independent experiments, each in triplicate).

### High-throughput compatibility of the DAB-ODC fluorescence assay

To date, pharmacological inhibition of ODCs has relied largely on substrate or product analogs, underscoring the need for assays that enable discovery of chemically distinct ODC inhibitors. A robust, quantitative, and scalable assay is therefore essential for both mechanistic studies and inhibitor discovery. Having established the performance of the DAB-based fluorescence assay using purified yeast and human ODCs, we next evaluated its suitability for high-throughput screening.

The amenability of the DAB-ODC assay to high-throughput formats was assessed by determining the signal-to-background (S/B) ratio, CV, and Z′ factor for ScSpe1, as described in the Experimental procedures. Assay performance was evaluated using the Z′ factor calculated from positive (fully active enzyme) and negative (complete inhibition) controls. Under these conditions, the assay yielded a Z′ factor of 0.86, with CV of 2.50% and 4.63% for active and inactive controls, respectively, and a S/B ratio of 4.18. To assess assay performance under inhibitor conditions, Z′ was also calculated using partially inhibited reactions (DFMO at 2.5 μM, ∼50% activity) relative to complete inhibition. Under these conditions, the Z′ factor remained high (0.72), with CV values of 4.33% and 4.63% and an S/B ratio of 2.76. These results demonstrate that the assay maintains robust performance across both maximal and intermediate activity ranges, supporting its suitability for high-throughput screening and quantitative inhibitor analysis.

Because chemical screening libraries are typically formatted in DMSO, we also evaluated the compatibility of the DAB–ODC assay with DMSO. Previous fluorescence-based ODC assays have been reported to suffer from DMSO-dependent quenching of fluorescence signals (33), limiting their utility for compound screening. In contrast, the DAB-based assay showed no measurable loss of fluorescence signal for either putrescine–DAB or ornithine–DAB adducts in the presence of DMSO concentrations up to 2.9%, corresponding to ∼10% DMSO in the ODC enzyme reaction prior to detection, well above the ≤1% DMSO typically used in screening assays. (Fig. S4). These results demonstrate that the DAB–ODC assay is tolerant to DMSO at concentrations relevant to high-throughput chemical screening.

Together with its non-radioactive format and quantitative reporting of ornithine-to-putrescine conversion, this assay provides a practical platform for kinetic analysis, inhibitor validation, and large-scale chemical screening aimed at identifying novel, non-analog modulators of ODC activity.

## Discussion

ODC occupies a central position in polyamine metabolism by catalyzing the rate-limiting conversion of ornithine to putrescine (4, 18). Dysregulation of ODC activity is strongly associated with uncontrolled cell proliferation, and elevated ODC expression and activity are well documented in a wide range of human cancers (10, 34, 35, 36). In addition, ODC is essential for the survival and virulence of multiple parasites, including kinetoplastids, and apicomplexans, making the enzyme an attractive therapeutic target for both oncology and antiparasitic drug development (10, 37, 38, 39, 40, 41). Despite this importance, the clinical and experimental ODC inhibitor landscape remains limited, with DFMO, an irreversible ornithine analog, being the only well-established inhibitor in widespread use (31). The scarcity of potent, non–substrate-analog ODC inhibitors underscores the need for new assay platforms capable of supporting large-scale discovery efforts.

In this study, we describe a fluorescence-based assay that directly reports ODC activity using DAB and βME to detect putrescine formation. The assay exploits the differential chemical reactivity of primary amines toward DAB, which is strongly influenced by the presence of an adjacent α-carboxyl group. Ornithine, a non-proteinogenic amino acid, contains two primary amines, but only one is distal from the α-carboxylated carbon, whereas putrescine contains two primary amines not associated with a carboxyl group. As a result, ornithine and putrescine form fluorescent DAB adducts with markedly different efficiencies, producing a robust fluorescence contrast that directly reports enzymatic decarboxylation (Fig. 1*C*). The addition of βME substantially enhances signal intensity, improving assay sensitivity, and enabling detection of subtle changes in enzyme activity.

Orthogonal validation using TLC supports that the fluorescence signal accurately reflects conversion of ornithine to putrescine by *S*. *cerevisiae* Spe1 and human ODC enzyme. The chemical identity of the fluorescent DAB–polyamine adducts has been established previously by mass spectrometry. Prior work identified the DAB–putrescine adduct as a substituted isoindole derivative (28), consistent with earlier mass spectrometric characterization of DAB adducts formed with other primary amines, ethanolamine (25). Differences in fluorescence intensity among DAB–ornithine and DAB–putrescine correlate with the number and accessibility of reactive amine groups. Together, these studies support the interpretation that the fluorescence observed here arises from formation of DAB–putrescine isoindole adducts generated downstream of ODC-catalyzed decarboxylation. Enzyme kinetics study of the ODC enzymes demonstrates that the apparent *K*_*m*_ value determined for ScSpe1 falls within the range reported for ODC enzymes from other organisms (42, 43). Mammalian ODCs typically exhibit *K*_*m*_ values for ornithine in the range of ∼ 60 to 100 μM, whereas ODCs from protozoan parasites and fungi often display higher *K*_*m*_ values, frequently between ∼120 and 300 μM depending on species and assay conditions (37, 44). The ScSpe1 *K*_*m*_ of ∼162.3 μM is therefore consistent with values reported for fungal and parasitic ODCs (42, 44), and is modestly higher than those reported for human ODC, reflecting potential species-specific differences in substrate affinity and regulatory architecture. These comparisons indicate that the kinetic parameters measured using the DAB-based fluorescence assay are physiologically reasonable and align well with values obtained using orthogonal assay formats, supporting the validity of this method for quantitative enzymology.

Importantly, the DAB-based assay is readily adaptable to high-throughput screening formats. Using DFMO as a benchmark inhibitor, we demonstrated that the assay can reliably quantify ODC inhibition and determine inhibitory potency. We further validated the platform using additional, structurally distinct ODC inhibitors with reported nanomolar activity, confirming that the assay sensitively detects inhibition across multiple chemical scaffolds. While DFMO has proven clinical utility, particularly in parasitic diseases, its mechanism as a suicide inhibitor and structural similarity to ornithine limit its applicability and motivate the search for chemically distinct, reversible, and more potent inhibitors. The ability of the DAB-based assay to directly measure product formation, without reliance on coupled reactions or labeled substrates, makes it particularly well suited for unbiased screening of diverse chemical libraries.

In conclusion, we present a sensitive, specific, and scalable fluorescence assay for measuring ODC activity that addresses a critical bottleneck in polyamine-targeted drug discovery. By enabling high-throughput identification of non–substrate-analog ODC inhibitors, this platform provides a valuable tool for advancing therapeutic strategies against cancer and parasitic infections. Future applications of this assay will facilitate systematic exploration of ODC inhibition and accelerate development of novel modulators of polyamine metabolism.

## Experimental procedures

### Materials

The human *ODC* (HuODC) sequence was codon-optimized for expression in *Escherichia. coli*, chemically synthesized and cloned into pMAL-c4x-1-H(RBS) plasmid by GenScript. The *S. cerevisiae* Spe1 expression plasmid (ScSpe1-pET-20b(+)) was obtained from Addgene (plasmid #117145), originally deposited by Markus Ralser (45). Putrescine (P5780-5G), L-Ornithine (O2375), 1,2-diacetylbenzene (242039-100MG), and Pyridoxal 5′-phosphate hydrate (P9255-1G) were purchased from Millipore Sigma. β-Mercaptoethanol (1610710) was purchased from Bio-Rad. DFMO was generously provided by the laboratory of Dr Robert Casero. Other ODC inhibitors, including 1-amino-oxy-3-aminopropane (APA), R-AOMP (10-R) and S-AOMP (10-S), were synthesized as previously described (31).

### Expression and purification of His6-tagged SPE1 and MBP-fused HuODC

The MBP-tagged HuODC expression plasmid (HuODC–pMAL-c4x) was transformed into *E. coli Rosetta (DE3)* cells. Cultures were grown in Luria–Bertani medium supplemented with ampicillin (50 μg/ml) at 37 °C to mid-log phase (OD_600_≈ 0.6), induced with 0.5 mM isopropyl β-D-thiogalactopyranoside, and incubated at 16 °C for 12 to 16 h. Cells were harvested by centrifugation and resuspended in lysis buffer containing 25 mM Tris-HCl (pH 8.0), 500 mM NaCl, 0.5% glycerol, 50 mM L-arginine, DNase I, protease inhibitor cocktail, and 0.002% CHAPS. Cells were lysed by sonication on ice, and insoluble material was removed by centrifugation at 16,000×*g* for 20 min.

The clarified lysate was incubated with amylose resin equilibrated in column buffer (20 mM Tris-HCl, 200 mM NaCl, 1 mM EDTA, 1 mM DTT) at 4 °C with gentle agitation. After extensive washing, maltose-binding protein–fused human ODC was eluted with a column buffer containing 10 mM maltose. Protein purity was assessed by sodium dodecyl sulfate–polyacrylamide gel electrophoresis (SDS–PAGE) with Coomassie staining, and protein concentrations were determined spectrophotometrically.

### Expression and purification of recombinant ScSpe1

The His_6_-tagged *S*. *cerevisiae* ODC (ScSpe1) expression plasmid (ScSpe1–pET-20b(+)) was transformed into *E. coli* Rosetta (DE3) cells. Cultures were grown and harvested by centrifugation as described above and resuspended in Ni-NTA lysis buffer (25 mM Tris-HCl, pH 8.0, 300 mM NaCl, 10 mM imidazole, 5% glycerol, DNase I, and protease inhibitor cocktail). Cells were lysed by sonication on ice, and clarified lysates were obtained by centrifugation at 16,000×*g* for 20 min. The supernatant was applied to Ni^2+^-charged affinity resin equilibrated with lysis buffer. The column was washed extensively with wash buffer containing 20 mM imidazole to remove nonspecifically bound proteins, and His_6_-ScSpe1 was eluted with buffer containing 250 mM imidazole. Eluted fractions were analyzed by SDS–PAGE with Coomassie staining to assess purity, and protein concentrations were determined spectrophotometrically.

### Fluorescence based DAB-ODC assay

The ODC enzyme reaction and subsequent detection of the products (putrescine) using 1, 2 DAB/βME were performed in the following sequential steps. In the first step, recombinant ScSpe1 and HuODC were used in the ODC assay. ODC enzyme reactions were performed in a final volume of 100 μl. Reactions were assembled using 50 μl of a 2× ODC enzyme mix prepared in buffer containing 0.2 mM EDTA, 0.2 mM pyridoxal 5′-phosphate, 5 mM DTT, and 50 mM Tris-HCl (pH 7.5). For inhibitor studies, 10 μl of inhibitor solution (DFMO or other compounds at the indicated concentrations) or 10 μl of water (no-inhibitor control) was added to the enzyme mix and incubated at room temperature for 5 min to allow inhibitor binding. Enzyme reactions were initiated by addition of 40 μl of L-ornithine solution (prepared in water), yielding a final ornithine concentration of 250 μM, unless otherwise specified. Reactions were incubated at 37 °C. At defined time points (0, 20, 40, 60, and 80 min), 17.5 μl aliquots were removed and immediately quenched by heating at 95 °C for 5 min. Samples were cooled on ice prior to analysis using the DAB-based fluorescence detection assay. Subsequently, 17.5 μl of each quenched enzyme reaction was combined with 17.5 μl water and 85 μl detection buffer (1.75 mM βME, 20 mM sodium buffer (pH 9.6), 0.22 mM potassium phosphate buffer, 1.48 mM 1,2 diacetylbenzene) in a 96-well black clear bottom plate (265,301, Thermo Fisher Scientific) and allowed to incubate at room temperature for 60 min. Following this, total fluorescence intensities (λ_ex_= 364 nm and λ_em_= 425 nm) were measured using a plate reader (Biotek Synergy H1, Agilent) and data were analyzed in GraphPad prism.

### Calculation of ODC activity and reaction velocity

Fluorescence intensities obtained from the DAB-based ODC assay were first corrected for background signal by subtracting fluorescence measured from mock reactions containing heat-inactivated enzyme in the absence of ornithine. To account for nonenzymatic fluorescence arising from direct reaction of ornithine with the DAB detection reagents, net fluorescence values were further corrected by subtracting the fluorescence measured from heat-inactivated enzyme reactions incubated in the presence of ornithine.

For time-course experiments, corrected fluorescence values were converted to percentage conversion of ornithine to putrescine by normalizing each value to the fluorescence signal obtained from a putrescine-only control, which was defined as 100% conversion under identical detection conditions.

For kinetic analyses, reaction velocities were calculated by converting normalized fluorescence values to micromolar concentrations of putrescine using the corresponding 100% putrescine control matched to each ornithine concentration. Velocities were expressed as μM putrescine formed per minute and plotted as a function of ornithine concentration. Data were fitted to the Michaelis–Menten equation by nonlinear regression using GraphPad Prism.

For inhibitor studies, enzyme activity was expressed as a percentage of the no-inhibitor control and used for comparative analysis of inhibition profiles.

### Assay considerations

Compounds containing primary amines may, in principle, react with DAB and contribute to background fluorescence. However, under the assay conditions used here, this contribution is minimal due to the low concentrations of inhibitors relative to enzymatically generated putrescine and the use of background subtraction based on heat-inactivated enzyme controls. In addition, any fluorescence arising from inhibitor–DAB interactions would not scale with enzyme activity and therefore does not affect the quantification of enzymatic conversion. Not all primary amines react efficiently with DAB under these conditions. In particular, amino acids, despite containing primary amine groups, do not produce measurable fluorescence in the DAB detection reaction, likely due to steric hindrance (25, 29). Similarly, millimolar concentrations of Tris buffer, pH7.4 carried over into the detection reaction did not produce measurable fluorescence (Fig. S5). Consistent with this, inhibition by DFMO and APA, both of which contain primary amine, was readily detected using this assay.

### Analysis of ornithine and putrescine by TLC

Putrescine formation from ornithine substrates after ODC reaction was confirmed by TLC on Silica 60 plates (Merck; 500 μm) using solvent system consisting of Chloroform: Methanol: Ammonia (2:2:1, v/v/v). Ornithine and putrescine were visualized with ninhydrin spray (0.2% in ethanol/acetic acid, 99.5/0.5) followed by incubation at 110 °C for 5 min.

### Data analysis to determine *Z*′ score

S/B ratios for ODC assays were calculated by dividing the mean fluorescence intensity of reactions containing active enzyme by the mean fluorescence intensity of reactions containing heat-inactivated enzyme. Assay variability was assessed by calculating the CV, defined as the standard deviation of the fluorescence intensity divided by the corresponding mean fluorescence intensity and expressed as a percentage. The Z′ factor, which integrates assay signal dynamic range and variability, was calculated using the following equation:

$$Z’=1-3\left(\sigma_{p}+\sigma_{n}\right)/\left|\mu_{p}-\mu_{n}\right|$$

where *μ*_*p*_ and *μ*_*n*_ are the mean fluorescence intensities of the positive (active enzyme, either fully active in the absence of inhibitor or partially active under 50% inhibition by DFMO) and negative (heat-inactivated enzyme) controls, respectively, and *σ*_*p*_ and *σ*_*n*_ represent the corresponding standard deviations. All values were calculated from replicate wells pooled across three independent plates.

## Data availability

All data are contained in the article and the supplemental data files.

## Supporting information

This article contains supporting information.

## Conflict of interest

The authors declare that they have no conflicts of interest with the contents of this article.

## Supporting information

Sup Figures
