# Supplementary material for: A direct fluorescence assay for quantitative analysis of ornithine decarboxylase activity and inhibitor screening
Source: J Biol Chem. 2026 Apr 17;302(6):111468. doi: 10.1016/j.jbc.2026.111468 (PMC13214536; doi:10.1016/j.jbc.2026.111468)
Supplement: Sup Figures [file mmc1.docx]

**Figure S1. Thin-layer chromatography analysis of the ornithine and putrescine mixture** Mixtures containing ornithine and putrescine at a constant combined concentration of 250 µM were analyzed by thin-layer chromatography. In each mixture, increasing amounts of putrescine were accompanied by corresponding decreases in ornithine. Spots were visualized using ninhydrin staining.

**Figure S2**. **Kinetic characterization of ScSpe1 and HuODC using the DAB-based fluorescence assay.** Net fluorescence generated by purified His₆-tagged S*. cerevisiae* Spe1 (ScSpe1) (**A**) and MBP-fused human ODC (**B**) measured using the DAB-based assay across increasing concentrations of ornithine (0–500 µM). Fluorescence increased with substrate concentration and reached saturation at approximately 250 µM ornithine, indicating enzyme saturation under these conditions. (B)

**Figure S3.** **Dose–response inhibition of ScSpe1 and HuODC by non-DFMO ODC inhibitors.** (**A–C**) Inhibition of *S. cerevisiae* Spe1 (ScSpe1) by APA (**A**), R-AOMP (**B**), and S-AOMP (**C**).(**D–F**) Inhibition of human ODC (HuODC) by APA (**D**), R-AOMP (**E**), and S-AOMP (**F**). ODC enzyme reactions were performed using 160 µM ornithine for ScSpe1 and 100 µM ornithine for HuODC. Reactions were incubated for 30 min at 37 °C in the presence of increasing concentrations of inhibitors (0–50 µM), as described in the Experimental Procedures. Reactions were terminated by heat inactivation, and ODC activity was quantified using the DAB-based fluorescence detection assay. Fluorescence signals were normalized to no-inhibitor controls and fitted by nonlinear regression to determine IC₅₀ values.

**Figure S4**. **DMSO compatibility of the DAB-based fluorescence assay.** Fluorescence signals generated by DAB adducts of putrescine and ornithine were measured in the presence of increasing concentrations of dimethyl sulfoxide (DMSO). No measurable reduction in fluorescence intensity was observed for either putrescine–DAB or ornithine–DAB adducts at DMSO concentrations up to 2.9%, indicating that the DAB-based assay is tolerant to DMSO at levels relevant to high-throughput chemical screening.

**Figure S5.**


**Figure S5 Effect of Tris on background fluorescence in the DAB-based ODC assay.** Background fluorescence was measured using mock ODC reaction mixtures containing Tris buffer, pH7.4 (3.5 mM final concentration) or water as a control. Aliquots (17.5 µL) were incubated with DAB detection buffer (102.5 µL), and fluorescence was measured as described in the Experimental Procedures. No detectable increase in fluorescence was observed in the presence of Tris.
